# An Online Survey of New Zealand Vapers

**DOI:** 10.3390/ijerph15020222

**Published:** 2018-01-29

**Authors:** Penelope Truman, Marewa Glover, Trish Fraser

**Affiliations:** 1 School of Health Sciences, Massey University, Wellington 6021, New Zealand; 2 Institute of Environmental Science and Research Ltd., Porirua 5240, New Zealand; 3 School of Health Sciences, Massey University, Auckland 0632, New Zealand; m.glover@massey.ac.nz; 4 Global Public Health Ltd., Glenorchy 9350, New Zealand; trish@tukuwahaglenorchy.com

**Keywords:** nicotine, electronic cigarettes, tobacco dependence, vaping, smoking cessation

## Abstract

Using electronic cigarettes (vaping) is controversial, but is increasingly widespread. This paper reports the results of an electronic survey of vapers in New Zealand, a country where the sale and supply of e-liquids containing nicotine is illegal, although vapers can legally access e-liquids from overseas. An on-line survey was conducted, using vaper and smoking cessation networks for recruitment, with follow up surveys conducted 1 and 2 months after the initial survey. 218 participants were recruited. Almost all had been smokers, but three quarters no longer smoked, with the remainder having significantly reduced their tobacco use. Three participants were non-smokers before starting to vape, but none had gone on to become smokers. The overriding motivation to begin and continue vaping was to stop or to reduce smoking. The results were consistent with a progression from initially both vaping and smoking using less effective electronic cigarette types, then moving to more powerful devices, experimentation with flavors and nicotine strengths—all resulting in reducing or stopping tobacco use. Lack of access to nicotine and lack of support for their chosen cessation method were the main problems reported. Vaping had resulted in effective smoking cessation for the majority of participants.

## 1. Introduction

Vaping (using an electronic vaporiser or e-cigarette (e-cig)) is a relatively new behavior that has spread throughout the world’s tobacco smokers [1,2,3]. The appeal of vaping, for people who either cannot or do not want to stop smoking, lies in reduced harm (and cost in some jurisdictions) [4,5,6] whilst maintaining a sufficiently similar, or superior, experience to smoking tobacco [7,8].

Each year, six million people are expected to die from smoking-related diseases [9]. Using e-cigs as a substitute for tobacco smoking has the potential to save many lives and reduce smoking-related morbidity [5]. However, as with many new technologies, e-cigs have been met with considerable skepticism and fear.

A wide range of claims supporting calls to ban or heavily restrict vaping have been made [10,11]. For example, it is suggested that vaping might increase youth initiation into smoking, renormalize smoking behavior, undermine smokefree goals [4,11], and that it may cause lung disease [12] and cancer [13]. However, contrasting research shows that people who switch from smoking to vaping exhibit health improvements [14], that youth smoking is declining where their access to e-cigs is not prohibited [15], and that toxicant levels in e-cig vapor are significantly lower than those from tobacco smoke [4,16]. While legitimate concerns remain [17,18], many initial fears appear to have little foundation [19,20,21].

Researchers and health professionals with concerns have lobbied for bans or heavy restrictions on vaping behavior, equipment, and products. Government, regulatory, and health authorities have tended towards restricting vaping, as a risk-avoidance response [22,23], until knowledge about vaping and other harm reduction alternatives to smoking grows.

Several surveys of vapers have been conducted internationally to determine the characteristics of vapers, types of e-cigs, strength of nicotine and flavors people were using and why [24,25,26], how long people vaped over time, if they had previously smoked tobacco, and if they had stopped or not [26,27]. Legislation to prohibit vaping varies greatly by country, and the policy environment may well affect who uses e-cigs, and why [28,29]. In many European countries, the USA, and Canada, initially vaping products were not restricted. Conversely, Australia and New Zealand applied existing legislation to prohibit the commercial import, sale, or distribution of nicotine-containing e-cigs and e-liquids (liquids used for vaping). Private importation is legal. The e-cigs themselves cannot be sold to anyone under 18 years of age and no therapeutic claims about e-cigs can be made. However, new legislation is under consideration and the New Zealand government has promised legislative change to allow the commercial import, sale, and distribution of nicotine-containing e-liquids [30].

Little is known about the perceptions and experiences of people who use e-cigs in countries with heavy restrictions on nicotine-containing e-liquids, vaping products, and vaping. Fraser et al. [31] have investigated Australian vapers and found that the majority were ex-smokers, now only vaping, whose major concern was ease of access to vaping products. Although some New Zealand surveys are starting to ask smokers whether they have tried vaping [1], little is known about the experiences of New Zealand vapers. We report here the results of an electronic survey of New Zealand vapers, (January to April 2016) part of a wider study to explore the ways these vapers use e-cigs and the effects of this use on themselves, and on those around them.

## 2. Materials and Methods

This was an exploratory on-line survey of people living in New Zealand who regularly used e-cigs. In-depth qualitative interviews of a randomly selected subset of participants were also conducted and are reported elsewhere (Fraser, Glover & Truman, in press).

The on-line questionnaire was designed to collect demographic information, smoking and dependency data, and information deemed important for the interviews with vapers. Survey questions were adapted from standard questionnaires [32,33] and using question content from the United Kingdom (UK) Royal College of Physicians and the National Institute of Health Innovation, University of Auckland. Detailed wording was moderated using feedback from knowledgeable vapers. 

An initial questionnaire gathered information on participants’ demographics, smoking and vaping histories, current consumption, as well as exploring problems encountered. The second and third questionnaires asked for updated information on the type of devices and e-liquid flavors being used, nicotine strength, whether the participant had smoked at all in the previous 24 h, and whether the amount they were vaping and the amount they were smoking had increased or decreased. In each survey, participants were given a range of specific options to facilitate classification, although free text options were included for some questions. An overview of the questionnaire information is shown in Table 1, with further details available in the Supplementary Materials, Files 1 and 2. (Questionnaire 1, File 1; Questionnaires 2 and 3, File 2). 

Research Electronic Data Capture (REDCap), a database linked to an on-line survey tool, was used to carry out the survey and was hosted by the University of Auckland (https://projectredcap.org).

We aimed to recruit 150 vapers via e-cig retailers, vaper groups and social media, and via links provided on the End Smoking NZ (www.endsmoking.org.nz) and Tobacco Control Research Tūranga (www.turanga.org.nz) websites. We contacted known e-cig retailers and asked if they would do the following: display an advertisement to recruit vapers to the study, alert potential participants about the study, and give us contact details of “vaper groups”. Twitter, New Zealand vaping Facebook pages, and blog spaces where vapers posted messages were utilized to advertise the study. Similar methods have proved successful in other countries with a low prevalence of vaping, e.g., [31].

Participants were shown participant information on-line and, once they had confirmed reading this information, a consent page was reached which required participants to give their informed consent in order to proceed to the survey. Participants were encouraged to ask the principal investigator any questions about the research. Participation in the study was voluntary and no inducements were offered.

Approximately 1 and 2 months after submission of the baseline survey, email notifications were sent to each participant who had provided their email address. The emails contained a unique URL that led respondents directly to the survey response web page to complete brief follow up surveys.

Ethical approval for the study was obtained from the Ministry of Health, Health and Disability Ethics Committee (Ethics Number 15/STH/215).

### Data Analysis

All respondents who had used e-cigs had consented to their information being included, had provided the majority of the information, and were from New Zealand, were included in the analysis. Baseline and follow-up data was exported to Excel and combined for analysis. For later analyses, participants were grouped into a “Vape only” group (reporting no cigarette use in any of the three surveys), and a “Vape and smoke” group, with two subgroups: “Occasional smokers” (non-daily smokers, reporting some smoking in any of the three surveys) and a “Vape and smoke daily” group (those who reported daily smoking at the first survey). For most characteristics, no between-group differences were observed and in this case, data from all participants was reported together. Statistical tests were performed either in Excel (Microsoft, Redmond, WA, USA), or in Graphpad Prism 7 (GraphPad Software, La Jolla, CA, USA) (non-parametric tests). 

Six participants were excluded from analyses of smoking and vaping characteristics; three who were no longer vaping at Questionnaire 1, and three who were non-smokers when they started vaping.

## 3. Results

There were 230 unique, completed, and consented initial questionnaires. 12 were excluded due to missing “region” information (*n* = 7; might not have been from New Zealand), or because they were definitely from overseas (*n* = 5). Of the remaining 218 questionnaires, 15 had some missing data. Two of the 218 gave no email address and could not be contacted for follow-up studies. Of the 216 participants invited to complete questionnaires 2 and 3, 157 (73%) completed the second, and the third questionnaire was completed by 118 (55%) participants (75% of those who filled in questionnaire 2). Only 25 (11%) had previously participated in other surveys on e-cigs.

### 3.1. Participant Characteristics

Gender, ethnicity, and age data for the participants is shown in Table 2, along with a summary of smoking history and vaping characteristics.

Participants ranged in age from less than 20 to over 65, with median age in the range 31 to 35. The participants were mostly male (77%) and of New Zealand Europeans (73%), although Māori (indigenous New Zealanders) were also well-represented, at 15%. Asian and “other” ethnic identities were better represented than those of Pacific origin, who were poorly represented in this survey (3 Pacific; 3 others of mixed ethnicity including Pacific). The highest number of participants (77, 35%) came from the Auckland region, New Zealand’s largest urban center, although participants from across the country took part.

All but 3 of the participants were regular vapers, who selected “usually vape every day” to describe their vaping. Of the three exceptions, two had vaped briefly but did not find it a good substitute for smoking. One had used vaping to quit smoking and had subsequently stopped vaping. These participants were included as being of interest to readers, but excluded from consideration of vaping and smoking characteristics, as not representing current vaper experience (the focus of this study). 

Overall, participants reported that they had been vaping from less than 1 month to over 2 years, with the majority (56%) having vaped for under a year. 

Of the 218 participants, 215 (99%) either were or had been smokers. Most (205, 94%) participants were smokers when they started vaping. Cigarettes smoked per day and the time to first cigarette after waking (TTFC), prior to initiating vaping, are shown in Table 3, showing that most of these participants were strongly tobacco dependent. 

### 3.2. Initiating Vaping

Participants could choose more than one reason for starting vaping. The most frequent reasons were to stop or cut down on smoking, including for health or financial reasons, at 83% overall. One participant noted “*My father died of lung cancer and I tried many methods to get off smoking. In the end vaping was almost an overnight transformation. I am almost at 1 year of no smoking, all due to vaping*”. This comment mirrored similar comments in other parts of the questionnaire.

Societal reasons (e.g., to use when socializing with smokers or to use where smoking was prohibited) were salient for 11% in total. Curiosity, or being interested in the technical side of e-cig use, was a factor for some (6%) (e.g., “*I enjoy tinkering so DIY vaping is right up my alley*”). 

The “other” category drew comments on the hobby aspect of vaping, concerns over reducing exposure of family members to cigarette smoke, or to deal with the triggers that might otherwise lead to smoking. 

Three participants reported being non-daily smokers when they started vaping. One commented “*I ‘socially’ smoked and very much enjoyed the act of smoking but did not want to become an everyday smoker*”.

### 3.3. Continuing Vaping

The single main reason for current vaping was “to avoid going back to smoking” (40%) with the next most frequent response being “I enjoy vaping” (24%). “*Vaping is superior to smoking in every single possible way. Cost, taste, health risk reduction and general pleasantness*”.

Health and cost reasons were also drivers for continued vaping. There were nine health-related comments including: “*My son (11) asked me to stop smoking ciggies because it stinks and I was having trouble breathing*”. Also, “*Vaping gives me the enjoyment of smoking with much less negative effects*”.

Societal reasons were less important, at under 1%.

### 3.4. E-Cigarette Type Used

Participants had mostly tried a wide variety of e-cig types, but the shift over time towards either tank or sub-ohm models was clear. Of the more powerful models, only about half of those who had tried the dripper type were still using them at the time of the survey. Disposable and rechargeable cigarette look-alike e-cigs were seldom still being used. The proportions of the types of e-cig in current use did not change between the three questionnaires.

### 3.5. Nicotine Strength

The most frequently reported nicotine strength was 1–6 mg nicotine/mL (Table 2). Twelve participants did not use nicotine at all, and a further 19 used e-liquids with no nicotine some of the time. Higher strength nicotine (>6 mg nicotine/mL) was used by 61 participants, of whom 47 used >6 mg/mL only. The proportion of participants using the different strengths of nicotine remained the same for the second and third questionnaires.

### 3.6. E-Liquid Consumption

The amount of e-liquid used per week varied widely, from <1 to 200 mL per week, with a median of 30 mL/week. Those who used the higher nicotine concentrations used lower volumes on average. The median volume for those who only used nicotine e-liquids at over 6 mg/mL was 15 mL per week. There were no trends in the amount of e-liquid used evident in the later surveys. 

### 3.7. E-Liquid Flavours

A wide variety of flavors was reported as being used, with most vapers (202, 93%) using several flavors, as shown by multiple listings and by changes in the flavor reported in successive questionnaires. Use of tobacco or menthol flavors was frequently (91, 42%) reported in answer to “what flavors have you used?”, but less frequently in answer to, “what flavors are you using now?” (22, 10%). This latter proportion was similar across the later surveys, and use of several flavors was still common.

### 3.8. Difficulties Experienced with Vaping

Almost half (46%) of the 287 responses reporting difficulties experienced with vaping concerned the availability of nicotine. The majority (82%) of participants reported one or more difficulties with the supply or quality of nicotine, or e-cigs and their components.

Of the 37 “other” problems, 17 specified that they had no problems with vaping. Ten of the comments were around price, availability, and quality of e-liquids and e-cigs, and three concerned the difficulties of accessing good information. Six people complained about the attitudes of others, e.g., The general public: “*People giving you shit about vaping, when they didn’t say anything when you combusted plant material*”.

Authorities: “*Bullshit attitudes from government*”.

Information sources: “*Outrageous claims surrounding the ‘dangers’ of vaping*”.

Prompted to report physical changes or symptoms, ten people responded, one of whom reported a positive effect. “*I have noticed from smoking my sense of taste/smell is poor, but is slowly coming back thanks to vaping*”.

Two people reported problems with bleeding or receding/sore gums, and another two reported that they had overdosed on nicotine initially and had needed to reduce the nicotine strength. Three people reported feelings of lung congestion, e.g., “*inhaling too much vapor and feeling like drowning*”. One person reported weight gain since taking up vaping, and one was concerned about spilling nicotine-containing e-liquid.

### 3.9. Support for Vaping

Support from other vapers, rated on a sliding scale, was perceived to be high, with a median of 99%, and a range of 50% to 100%. The range of support experienced from smokers and non-smokers varied widely ranging between 0% and 100%, with smokers being possibly a little more supportive (median 72%) than non-smokers (median 67%).

Individual vapers had had very different experiences, with some reporting good support from smokers and negativity from non-smokers, and vice-versa. Twenty-seven (12%) vapers in this survey rated the support they received at under 20% for one or more of these groups.

### 3.10. Smoking Status and Current Vaping

Four groups of vapers emerged from the data.

#### 3.10.1. Vapers Who No Longer Smoke (Vape Only Group)

The majority of participants (159, 73%) were smokers when they started vaping, and now only vaped. One had subsequently stopped vaping. All considered that their vaping and their stopping smoking were related. The majority (79%) said that they had used vaping to help them stop smoking. Twenty (12%) reported that they had not intended to stop smoking when they started to vape. The remainder had used vaping to avoid relapse to smoking.

#### 3.10.2. Continuing Smokers Who Both Vape and Smoke (Vape and Smoke Group)

51 vaping participants (23%) reported some smoking during the course of these surveys indicating that they were both vaping and smoking. Two of these came from the ex-smoker group. Of these 51, 34 (67%) only smoked occasionally (less than once a day). There were no apparent differences in demographic characteristics, in types of equipment used, the proportion of tobacco flavors used, and in the problems and attitudes experienced between those who only vaped and those who both vaped and smoked, whether smoking was regular, or not.

Both smokers and non-smokers cited “to avoid going back to smoking” as the most common reason for continuing to vape. However, unlike the “Vape only” group where 29% (95% CI; 23–38%) said that the main reason they still vaped was that they enjoyed vaping, only one of the group who still smoked (95% CI; 0–18%) reported this as the major reason for continuing vaping. Other differences were seen in a higher proportion of participants intending to cut down, rather than stop smoking, amongst the continuing smokers (95% CI; 12–31%) than in the “Vape only” group (95% CI; 5–9%). A quarter (13) cited “to stop smoking” as the main reason to continue vaping.

#### 3.10.3. Ex-Smokers Who Now Vape

10 participants (5%) were ex-smokers when they started to vape. Of these, seven cited “to avoid relapse to smoking” as the reason for vaping. Three of these used no nicotine. Two participants both smoked and vaped, using vaping as a way to cut down on the amount they smoked at relapse. Although these two participants did use nicotine, they were using very little e-liquid (10 and 2 mL/week, respectively). Neither of them completed questionnaires 2 or 3 so we cannot assess whether they continued vaping and/or smoking or not. One further participant cited “curiosity” as the reason they tried vaping.

#### 3.10.4. Non-Smokers Who Starting Vaping

Three vapers (1%) classified themselves as never having been a smoker. Two cited “curiosity” for the reason they started to vape, and two were vaping when socializing with other vapers, one writing “*to support my friend to stop smoking*” as her reason to start vaping. One enjoyed the technical side of vaping. All three had been vaping over 6 months at the start of the study and were between 20 and 40 years old. Two had used a disposable e-cig and one a pen-type e-cig at some stage and all were using more advanced types of e-cig by the time they filled in questionnaire 1, with nothing less than a tank system being currently used. All three checked both “no nicotine” and “1–6 mg/mL” as the strength of nicotine currently used, and all used 10–30 mL of e-liquid per week (within the lower half of users). Two of the three filled in questionnaires 2 and 3, and neither had used tobacco at any stage.

In addition, three participants were no longer vaping at the start of the study. One had used vaping to give up smoking and had then stopped vaping. Two participants reported that vaping did not work for them. In both cases, this happened quite early on (length of time vaping <1 month) and in neither case had a more modern e-cig type, with improved nicotine delivery, been used.

A summary of these groups and the interchanges between them is shown in Supplementary Materials File 3.

### 3.11. Smoking Cessation and Reduction in Smoking

Table 4 shows the reduction in smoking for all those who were smokers at the time they took up vaping. No differences were found between the two “Vape and smoke” subgroups in the characteristics reported in Table 4.

In addition to the majority, who had largely or completely stopped smoking, the current smokers had also cut down markedly on their tobacco consumption. Almost two thirds (63%) reported smoking only occasionally. Nevertheless, nearly 10% of participants (17) were both smoking regularly and vaping at the time of the first survey, having cut their tobacco use by approximately half (data not shown). Later surveys suggest a further decline in smoking with time (Table 5).

### 3.12. Dependence on Vaping

For some participants, there was no change in how soon after waking they had smoked their first cigarette and how soon after waking they now vaped (Table 4). A few were vaping sooner after waking than they had smoked, but over half (106; 53%) were vaping later after waking than they had smoked. The change in this dependence-related parameter was significant (Wilcoxon paired signed ranks test; *p* < 0.001). 

The results for dependence-related parameters by group can be seen in Table 6. The subgroup who only smoked occasionally used lower volumes of e-liquid (*p* < 0.01, Kruskal Wallace test), with a median of 15 mL of e-liquid per week compared to 30 mL and 31.5 mL for the “Vape only” and “Vape and smoke daily” groups, respectively. Furthermore, the “Vape only” group had a lower proportion using nicotine in the 7–12 mg/mL range compared with the two smoking subgroups (chi-square test; *p* = 0.049). 

The distribution of TTFV was similar between “Vape only” and the subgroups who also smoked, and there were no significant differences between groups in the profile of tobacco dependence before vaping started, as judged from the TTFC, and the number of cigarettes smoked.

A clear trend was, however, seen in the years each group had been vaping, with the “Vape only” group tending to have vaped for longer than the groups who were smoking (Table 6) (chi-square test; *p* < 0.01).

## 4. Discussion

This is the first comprehensive survey of New Zealand vapers, sampling a broad range of vapers over a short time-frame. New vapers were represented within the participant group, where a third had been vaping less than 6 months, and the majority, less than a year. The participants came from across the country and a range of ages and ethnicities, but represented only a small sample of the approximately 63,000 daily vapers in New Zealand [1].

The majority of participants were regular vapers who had switched completely from smoking by the time of these surveys (158 of 218).

Overwhelmingly, vaping was a means of cessation, smoking reduction, or avoidance/mitigation of relapse to smoking. However, because of the short duration of the project, it is unclear whether some of the “Vape and smoke daily” and the “Occasional smoke” groups may have been in the process of relapse to smoking, or transitioning towards only vaping [27].

The information given was consistent with a view that vapers tended to start with models that looked like a cigarette, but moved on to explore more powerful models, finding tank and sub-ohm models the most satisfactory. They also moved away from tobacco and menthol flavored e-liquid, over time.

Participants who both vaped and smoke tended to be newer vapers, consistent with a pattern where vapers may both smoke and vape initially, but will reduce their exposure to tobacco over time. The majority (66%) of those who still smoked only did so occasionally, and those who still smoked regularly had reduced their tobacco consumption by around 50%, on a conservative estimate. This level of reduction in smoking is consistent with overseas findings for dual users [27,34].

This proportion of participants both smoking and vaping (24%) is similar to that found in some studies [4,35], but is lower than the 50% cited by others, e.g., [36]. Some of this discrepancy may be due to differences in the definition of “dual use”, but the lack of representation, in this survey, of vapers who mostly smoke but substitute an e-cig for smoking only occasionally (regular weekly/monthly vapers) will have affected this proportion.

As yet, there are no well-validated measures of dependence in vapers. By analogy with smokers, two key measures to look at would be the amount of nicotine consumed, and the time to first vape on waking. Clear increases in time to first vape on waking compared with the time to first smoke on waking suggested a decrease in dependence. However, it was not possible to accurately establish nicotine intake from these questionnaires.

The later surveys were consistent with a continuing reduction in smoking and dependence, but should not be over-interpreted, as attrition bias may have influenced this result (see Supplementary Materials File 3). They are included for completeness.

There were very few adverse effects of vaping reported (2 × gum problems, 3 × congestion, 2 × initial nicotine overdose, and 1 × weight gain). Weight gain is not usually seen when smokers switch to vaping, although it is common when people stop smoking [37]. Participants were not asked about the benefits of vaping, but the comments suggested that health benefits from switching to vaping were experienced by at least some participants.

The observed pattern of occasional smoking by those who were regular vapers, together with the prominence of “to avoid going back to smoking” as a reason given for continuing vaping, suggests that part of vaping’s importance may be in preventing relapse to smoking, as suggested by others [26]. This aspect of vaping deserves further investigation.

Unlike nicotine patches and other smoking cessation methods, vaping appears to be actively pleasurable for many, as also suggested by others, e.g., [8]. Finding a pleasurable form of vaping may prove important for complete smoking cessation, since those who were smoking as well as vaping seemed less likely to report that they liked vaping. Exploration of different types of e-cigarette, different nicotine strengths, and different flavors of e-liquid was common and may be an important aspect of a successful vaping experience.

The biggest difficulties that vapers experienced were in supply of nicotine, the quality of the equipment, and the advice and support available. Most had experienced negative attitudes towards vaping from some sectors of society.

The number of people taking up vaping without having been a smoker was very low (1.4%) in line with recent UK experience of vaping [15]. Overall, these data give no support to any concerns about widespread non-smoker initiation into vaping.

Overall, although this was a biased sample, weighted towards those vapers who were most committed to vaping, within that limitation, the results of this survey are supportive of a wider use of e-cigs for smoking cessation in New Zealand.

### Limitations of the Study

The sample was non-random, being based on recruitment through vaper networks. Within this population, the age and regional information of participants showed a good spread of participants, although women and people of Pacific ethnicity were under-represented. The participants in this survey would require access to computer technology and thus our recruitment path may have been weighted not only towards those in contact with active vaping communities, but also those of higher socioeconomic status.

The Health Promotion Agency’s figures on vaping [1] indicate that almost half of the regular adult vapers in New Zealand vape weekly or monthly, rather than daily, and this type of vaper has not been represented in this survey.

Those starting vaping, with nicotine, in New Zealand have to overcome barriers to do so, and may not be typical of vapers in countries where access to vaping is less restricted. Further differences between New Zealand and some other countries lie in high tobacco tax rates and strong societal pressures against smoking resulting from strong tobacco control policies. Thus, the findings from this study should be extrapolated to other jurisdictions with care.

Ex-vapers, stopping vaping because they had quit smoking successfully or because they had reverted to smoking, were not targeted in this survey, which aimed to explore the experiences of current vapers. These groups will not have been well-represented.

The survey concentrated on vaper perceptions of problems with vaping. As we did not ask about the positive benefits of vaping, we may have missed hearing of some of the positive aspects that these participants experienced, though some of these aspects will have been covered in the subsample selected for interview (reported elsewhere). 

The short duration of the survey and attrition rate meant that we could not effectively follow longer term trends. Further research is warranted, to follow the smoking cessation trajectory of a random sample of new vapers using vaping for smoking cessation, including much more detailed information on cigarette and e-cig use, and nicotine intake.

## 5. Conclusions

This survey of committed vapers suggests that, in New Zealand, with its advanced and highly dissuasive tobacco control program, vaping is almost exclusively used as a cessation tool. For many, it appears to have been successful.

The moves the New Zealand Government are now considering, to legalize the sale of e-liquid containing nicotine, will remove what vapers perceive as a key barrier to vaping. 

The planned legislative change may facilitate a positive shift in attitudes towards people who vape, encouraging people to persist with their smoking cessation goals and help New Zealand to reach its overall Smokefree 2025 goal of a smoking rate of less than 5%.

## Figures and Tables

**Table 1:** Questionnaire design *.

Questionnaire 1
**A: Vaping Patterns**
Length of time vaping
Types of e-cig used (past/present)
Vaping frequency, and volumes of e-liquid used
Strengths of nicotine (past/present)
Flavours used (past/present)
**B1: Vaping and Smoking History**
Reasons for vaping (past, present)
Problems experienced
Still vaping?
Still smoking?
**B2: Vaping Context**
Previous smoking amount
Current smoking amount
Time to first vape (present) and smoke (past)
Smoking/vaping balance
Reactions of others (smokers, non-smokers, vapers)
**C: Demographics**
Gender
Age
Ethnicity
Region
**Questionnaires 2 and 3 (Monthly Intervals)**
Type of e-cig used
Strength of nicotine used
Flavours used
Vaping increased/decreased over the last month?
Smoked at all in the last 24 h?
Smoking increased/decreased over the last month?
Smoke or vape first in the morning?
Time to first vape or smoke?

* For detail of question format, see Supplementary Materials.

**Table 2:** Summary of the characteristics of the survey participants (*n* = 218).

Characteristic	Number
**Gender** (*n* = 218)	
Male	168
Female	50
**Ethnicity** (*n* = 218: multiple responses allowed)	
Māori	32
Pacific	6
NZ European	174
Asian	13
Other	15
**Age** (*n* = 218)	
<20	5
20–30	87
31–40	61
41–50	45
51–60	8
>60	12
**Smoking and vaping history** (*n* = 218)	
Never smoker	3
Smoked at vaping start	205
Ex-smoker at vaping start	10
Vaping now (Questionnaire 1)	215
Smoking now (Questionnaire 1)	48
**Length of time vaping** (*n* = 217: 1 missing data)	
<1 year	121
1–2 years	47
>2 years	49
**Highest strength of nicotine used now** (*n* = 212: 3 “don’t know”, 3 ex-vaper)	
No nicotine only	12
1–6 mg/mL	139
7–12 mg/mL	39
13–18 mg/mL	15
>18 mg/mL	7
**Volume of e-liquid used** (*n* = 201: 14 missing data, 3 ex-vaper)	
Low (<10 mL/week)	20
Medium (10–35 mL/week)	112
High (35–70 mL/week)	58
Very high (>70 mL/week)	11

**Table 3:** Smoking characteristics of participants before taking up vaping (*n* = 205 *).

Time to First Cigarette on Waking	No: of Cigarettes/Day		
	1 to 10	10 to 20	>20
<5 min	4	46	21
6–15 min	17	43	14
16–30 min	7	20	3
31–60 min	6	5	2
Over an hour	9	3	1

* 10 ex-smokers, information not requested, 3 non-smokers.

**Table 4:** Changes in smoking for smokers taking up vaping: (*n* = 212) *.

Classification	Vape Only	Both Smoke and Vape
**Self-reported smoking before vaping started** (*n* =201; 1 missing data; 10 ex-smokers, no smoking history requested)		
Occasional (<1/day)	3	0
1–10	33	8
11–20	87	30
>20	34	6
**Total**	**157**	**44**
**Self-reported smoking at Questionnaire** 1 (*n* =212)		
None	166	0
Occasional (<1/day)	0	29
1–10	0	13
11–20	0	4
>20	0	0
**Total**	**166**	**46**
**Time to first cigarette on waking (TTFC) when smoking compared to time to first vape on waking (TTFV) once vaping** (*n* =200, 2 missing data; 10 ex-smokers, no smoking history requested)		
TTFV was shorter than TTFC	16	2
TTFV = TTFC	58	18
TTFV was longer than TTFC	82	24
**Total**	**156**	**44**

* 3 never-smokers and 3 who were no longer vaping were not included in this analysis.

**Table 5:** Tobacco use and first vape on waking (TTFV) reported in Questionnaires 2 and 3.

Classification	Vape Only	Both Smoke and Vape
**Self-reported smoking at Questionnaire 2 (3)** (*n* = 154 (118)) ^#1^		
None	123 (97)	11 (9)
Occasional (<1/day) ^#2^	2 (2)	8 (5)
1–10 ^#2^	2 (0)	7 (4)
11–20	0 (0)	0 (0)
>20	0 (0)	1 (0)
**Total**	**127 (99)**	**27 (18)**
**TTFV at Questionnaire 2 (3) compared with TTFV at Questionnaire 1**		
TTFV decreasing	15 (17)	7 (2)
TTFV the same	21 (50)	11 (10)
TTFV increasing	54 (29)	7 (6)
**Total**	**90 ^#3^ (96)**	**25 (18)**

^#^ Notes: ^1^ 3 never-smokers and 3 who were no longer vaping were not included in this analysis; ^2^ 5 participants, not smoking at questionnaire 1, reported some smoking in questionnaires 2 or 3; ^3^ Missing data: see supplementary information (Supplementary Materials File 2).

**Table 6:** Characteristics of vaper groups.

Category	Vape Only	Occasional Smoke	Vape and Smoke Daily
Number	161	34	17
**Vaping characteristics**			
**Years vaping**			
Under 1 year	81 (50%)	20 (61%)	15 (88%)
1 to 2 years	37 (23%)	10 (30%)	
Over 2 years	43 (27%)	3 (9%)	2 (12%)
**Strength of nicotine used (multiple responses possible)**			
No nicotine	15 (8%)	2 (5%)	2 (11%)
1–6 mg/mL	121 (66%)	20 (54%)	10 (53%)
7–12 mg/mL	25 (14%)	13 (35%)	5 (26%)
12–18 mg/mL	15 (8%)	2 (5%)	1 (5%)
>18 mg/mL	6 (3%)	0	1 (5%)
**Volume of nicotine used/week (mL)**			
Median	30	15	31.5
Interquartile range	20–50	10–30	15–62.5
**TTFV**			
0–15 min	68 (42%)	15 (47%)	5 (29%)
16–30 min	32 (20%)	7 (22%)	6 (35%)
31–60 min	41 (25%)	3 (9%)	4 (24%)
After an hour	19 (12%)	7 (22%)	2 (12%)
Not daily vaper	1 (1%)		
TTFC, before vaping started *			
0–15 min	112 (74%)	22 (67%)	10 (63%)
16–30 min	17 (11%)	8 (24%)	5 (31%)
31–60 min	11 (7%)	2 (6%)	
After an hour	10 (7%)	1 (3%)	1 (6%)
Not daily smoker	2 (1%)		

*n* = 212: 3 never smokers and 3 who were no longer vaping were not included in this analysis. * Details for 4 participants missing.

## Supplementary Materials

The following are available on-line at http://www.mdpi.com/1660-4601/15/2/222/s1, File 1: Questions from on-line Questionnaire 1, with selected data; File 2: Questions from 2^nd^ and 3^rd^ on-line questionnaires; File 3: Flow diagram showing vaping trajectories of participants.
