# Supplementary material for: An Online Survey of New Zealand Vapers
Source: Int J Environ Res Public Health. 2018 Jan 29;15(2):222. doi: 10.3390/ijerph15020222 (PMC5858291; doi:10.3390/ijerph15020222)
Supplement: Supplementary file 1 [file ijerph-15-00222-s001.zip › ijerph 254738 Supplementary Materials- File 2.pdf]

International Journal of
Environmental Research and Public Health

MDPI

Supplementary Materials: An Online Survey of New Zealand Vapers

Supplementary File 2: Questions from 2nd and 3rd on-line questionnaires.

1. What type(s) of e-cigarette are you vaping with? (Disposable cigarette lookalike, rechargeable cigarette look-alike, pen style, tank system, subtank, dripper, not applicable/not current vaper)
2. What strength(s) of nicotine do you currently have in your e-cigarette(s)? (No nicotine, 1-6 mg nicotine/mL, 7-12 mg/mL, 13-18 mg/mL, over 18 mg/mL, not applicable/not current vaper)
3. What flavour e-liquid(s) are you currently using? (Free text box, not applicable/not current vaper)
4. On average, has the amount you are vaping changed, compared to last month? (Less, about the same, more, none)
5. How many cigarettes did you smoke in the last 24 hours? (None, one cigarette, 1-10 cigarettes, more than 10 cigarettes, not applicable, not current smoker)
6. On average, has the number of cigarettes you smoke changed compared to last month? (Less, about the same, more)
7. In the morning, would you usually smoke first or vape first? (Smoke, vape, not applicable)*
8. How soon after you wake do you usually smoke or vape? (Within 5 minutes, 6-15 minutes, 16-30 minutes, 30- 60 minutes, after an hour)

* Note: In Questionnaire 2 the "not applicable" option in question 7 took participants to the end of the survey, if selected, and participants were not then asked for their time to first vape or smoke. This anomaly was removed for Questionnaire 3.
