# Supplementary material for: An Online Survey of New Zealand Vapers
Source: Int J Environ Res Public Health. 2018 Jan 29;15(2):222. doi: 10.3390/ijerph15020222 (PMC5858291; doi:10.3390/ijerph15020222)

International Journal of
Environmental Research and Public Health

MDPI

Supplementary Materials: An Online Survey of New Zealand Vapers

Supplementary File 1: Questions from on-line Questionnaire 1

A. **Could we confirm:** Have you ever used an e-cigarette? (Triage out if "No")
1. For about how long have you used or did you use an e-cigarette? (Less than 1 month, 1 to 3 months, 4-6 months, 7-12 months, 1-2 years, over 2 years)
2. Which of the following types of e-cigarette have you tried? Disposable cigarette look-alike, rechargeable cigarette look-alike, pen style, tank system, sub ohm, dripper)
3. Which of the following types of e-cigarette do you use now? Disposable cigarette look-alike, rechargeable cigarette look-alike, pen style, tank system, sub ohm, dripper)
4. How often do you vape? (Usually every day, less than daily, less than weekly but at least once a month)
5. What strength(s) of nicotine do you currently vape with? (No nicotine, 1-6 mg nicotine/mL, 7-12 mg/mL, 13-18 mg/mL, over 18 mg/mL, don't know)
6a. If you used pre-filled cartridges or disposable e-cigarettes, approximately how many would you use per week?<br>6b. If you use re-fillable systems, approximately how much e-liquid would you use per week (mL)?
7. How soon after you wake up do you usually vape? (Within 5 minutes, 6-15 minutes, 16-30 minutes, 31-60 minutes, after an hour, not applicable/not a daily vaper)
8. What flavour(s) of e-liquid are you using today?
9. What flavour of e-liquid did you use when you first started vaping?

For how long have you been vaping? (Qu. A1)

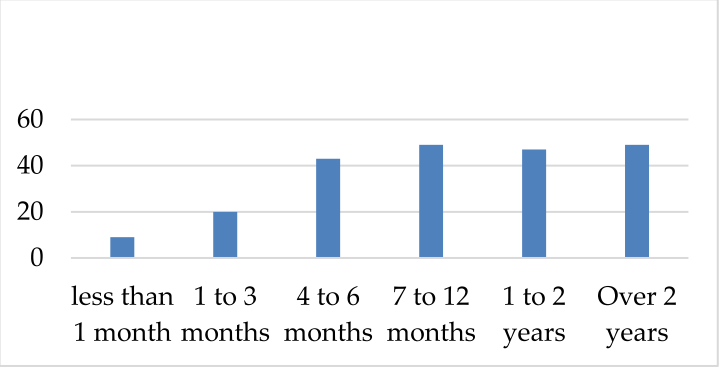

What types of e-cigarette have you used /do you used now? (Qu. A2/A3)

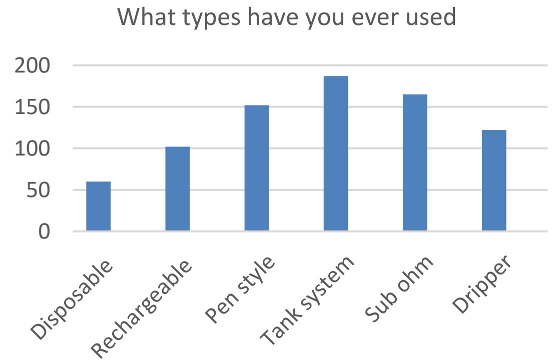

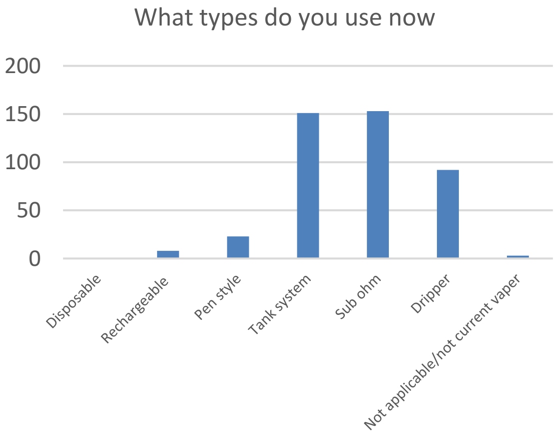

International Journal of
Environmental Research and Public Health

MDPI

What strength of nicotine do you currently use? (Qu. A5)

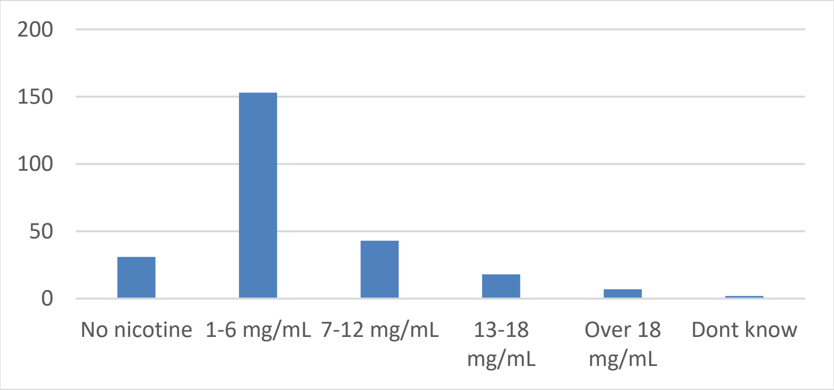

How much e-liquid would you normally use per week? (Qu. A6)

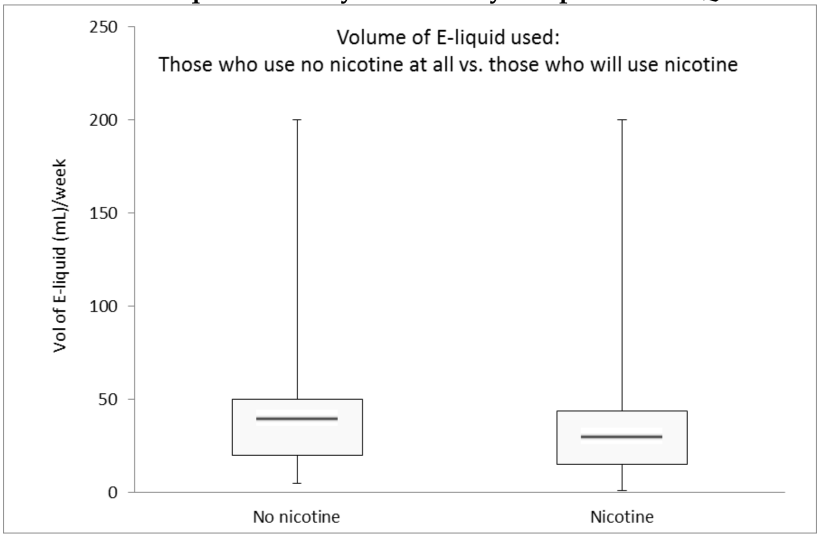

How soon after waking do you usually vape? (Qu. A7)

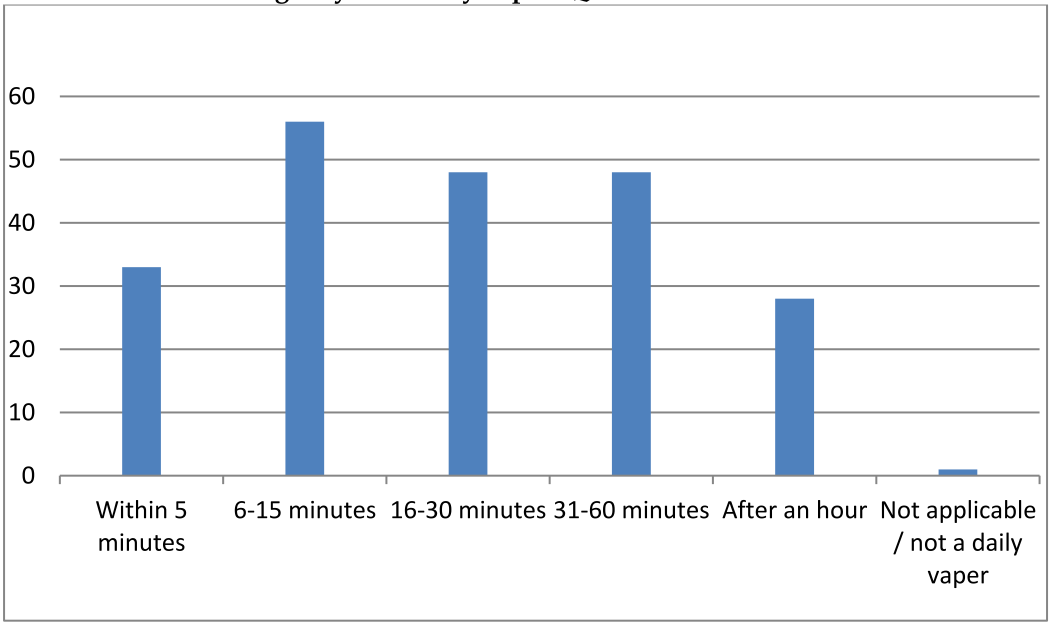

International Journal of
Environmental Research and Public Health

MDPI

B Please tell us a little about why you are using e-cigarettes, and your smoking history, if applicable:
1. Why did you start vaping? (Curiosity, to improve my health, to save money, to stop smoking, to avoid going back to smoking, for use in places where I cannot smoke, to use when socialising with smokers, to use when socializing with vapers, other)
2. What is the **main** reason you are vaping now? (To improve my health, to save money, to cut down on the amount I smoke, to stop smoking, to avoid going back to smoking for use in places where I cannot smoke, to use when socialising with vapers, I enjoy vaping, not applicable/not currently vaping, other)
3. What, if any, difficulties are you experiencing/have you experienced with vaping? (I do not enjoy vaping, not enough nicotine to satisfy cravings, physical changes or symptoms, battery goes flat too fast, e-cig breakdowns, difficulty obtaining e-cig replacements/parts, difficulty obtaining nicotine e-liquid, other)
4. Are you still vaping? (Yes, go to question B6; no)
5. Why did you stop vaping (Vaping did not relieve my cravings, I like smoking better, because of all the problems listed above, I no longer need to vape, other, go to question B9)
6. On a scale of 1-5 how supportive of you vaping have you found smokers to be? (1=not supportive, 3=don't care either way, 5=very supportive)
7. On a scale of 1-5 how supportive of you vaping have you found people who do not smoke to be? (1=not supportive, 3=don't care either way, 5=very supportive)
8. On a scale of 1-5 how supportive of you vaping have you found other vapers to be? (1=not supportive, 3=don't care either way, 5=very supportive)

Why did you start vaping? (Qu. B1)

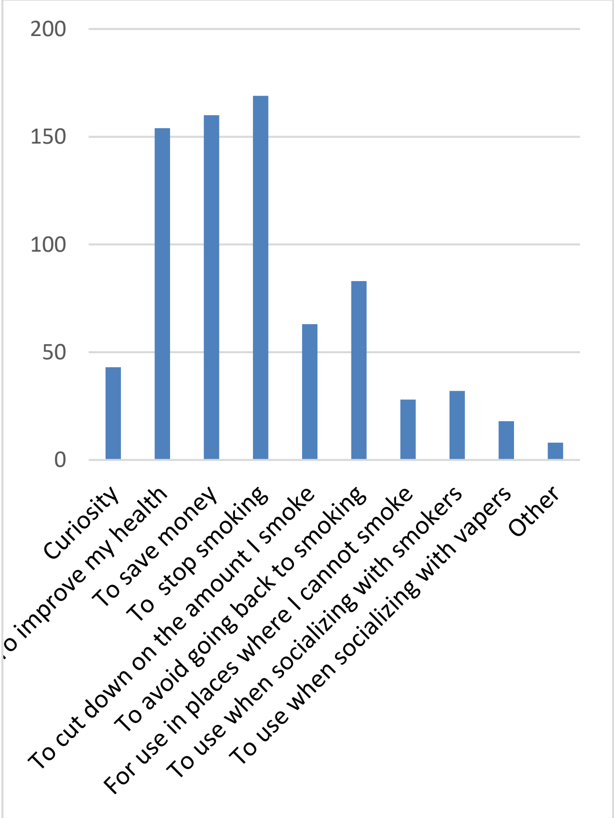

What is the main reason you are vaping now? (Qu. B2)

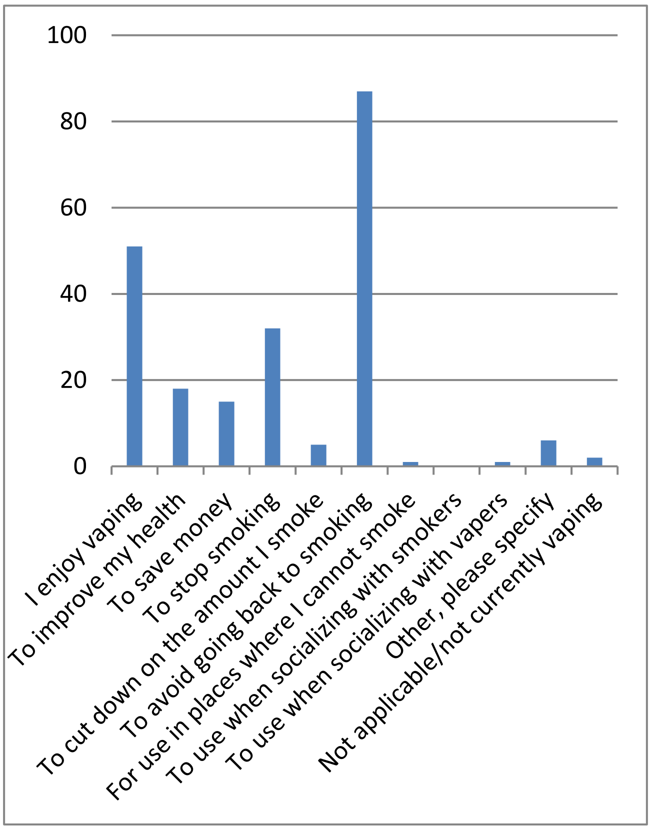

What difficulties have you experienced? (Qu. B3)

International Journal of
Environmental Research and Public Health

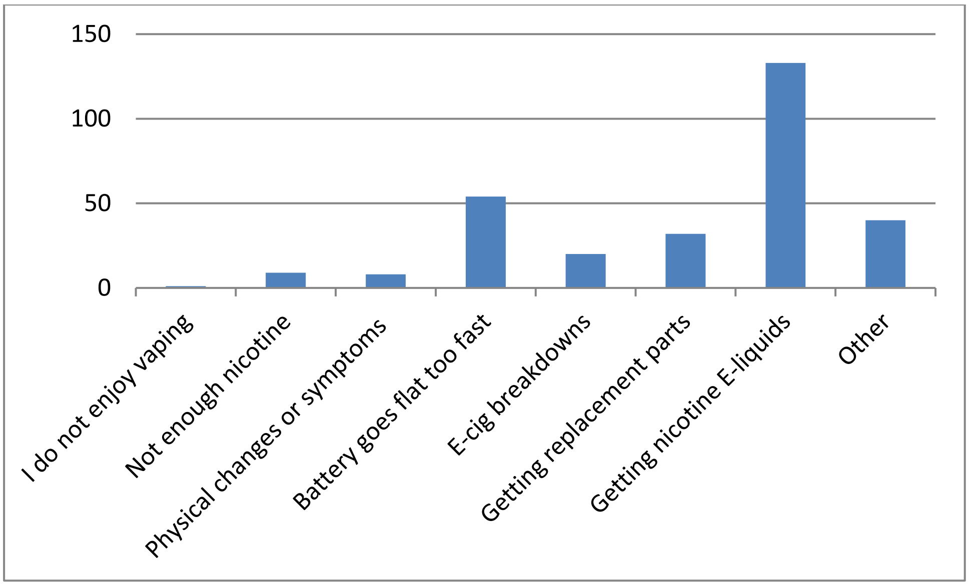

Why did you stop vaping? (three participants)

Vaping did not relieve my cravings
I like smoking better
I no longer need to vape

How supportive of vapers have you found others to be? (Qu. B6-B8)

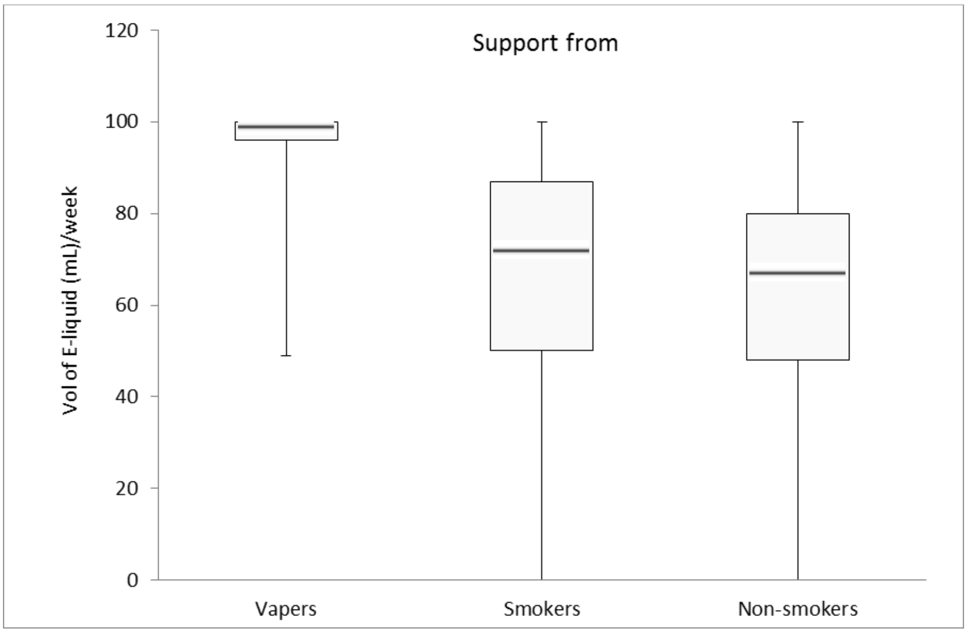

9. Are you, or have you ever been, a smoker? (Yes, no go to question C1)
10. Were you a smoker when you started to vape? Yes, no go to question B13)
11. When you were a smoker (before you started to vape) how many cigarettes did you usually smoke per day? (Occasionally, less than one cigarette a day, 1-10 cigarettes a day, more than 10 cigarettes a day)
12. How soon after waking did you usually smoke? (Within 5 minutes, 6-15 minutes, 16-30 minutes 30-60 minutes, after an hour, not applicable/not daily smoker)
13. How many cigarettes do you usually smoke now? (None, go to question 19; occasionally, 1-10 cigarettes a day, more than 10 cigarettes a day)
14. How soon after waking do you usually smoke? Within 5 minutes, 6-15 minutes, 16-30 minutes, 30-60 minutes
15. On a scale of 1-10 for an average day, would you mainly smoke or mainly vape? (1=mainly vape, 5=half & half, 10=mainly smoke)
16. On a scale of 1-5, to what extent is your decision whether to smoke or vape governed by where you are? (1=not at all, 3=somewhat, 5=completely)
17. On a scale of 1-5, to what extent is your decision whether to smoke or vape governed by who you are with? (1=not at all, 3=somewhat, 5=completely)
18. On a scale of 1-5, to what extent is your decision whether to smoke or vape governed by which you feel you would prefer? (1=not at all, 3=somewhat, 5=completely)
19. Which of these statements best describes your stopping smoking? (I used vaping to help me stop smoking, I used vaping to help me not start smoking again, my stopping smoking had nothing to do with my taking up vaping, I hadn't intended to stop smoking but I no longer smoke because I vape)
20. Have you taken part in any other New Zealand surveys about electronic cigarettes or vaping?

Cigarettes per day when vaping started (Qu. B11)

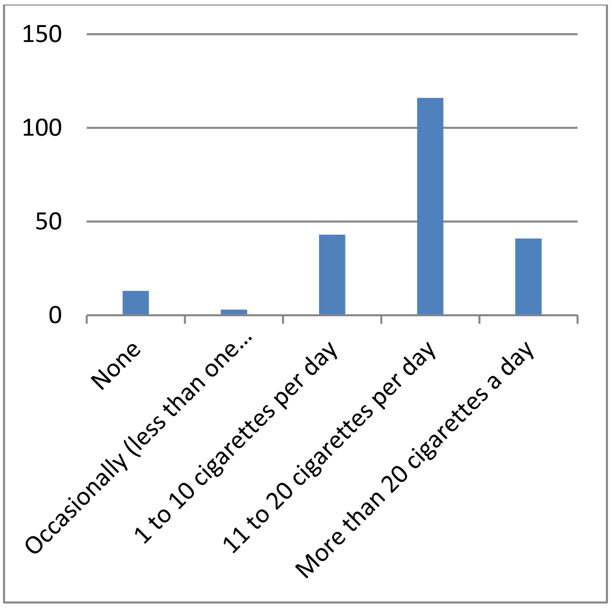

Cigarettes per day now (Qu. B13)

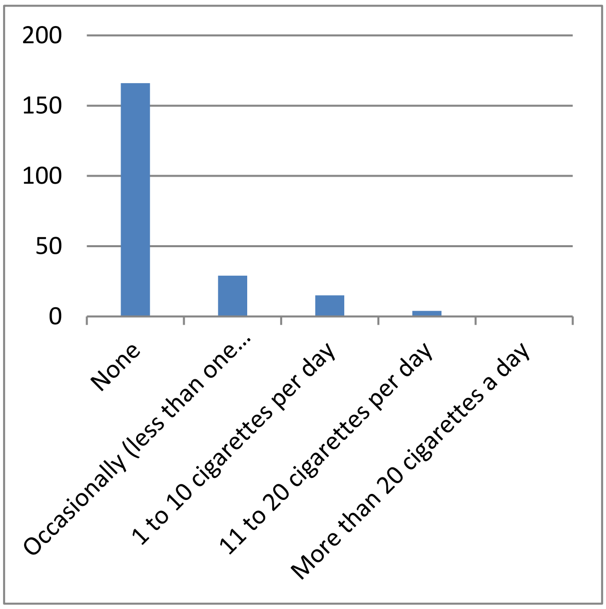

International Journal of
Environmental Research and Public Health

MDPI

How soon after waking did you usually smoke? (Qu. B13, compare Qu. A7)

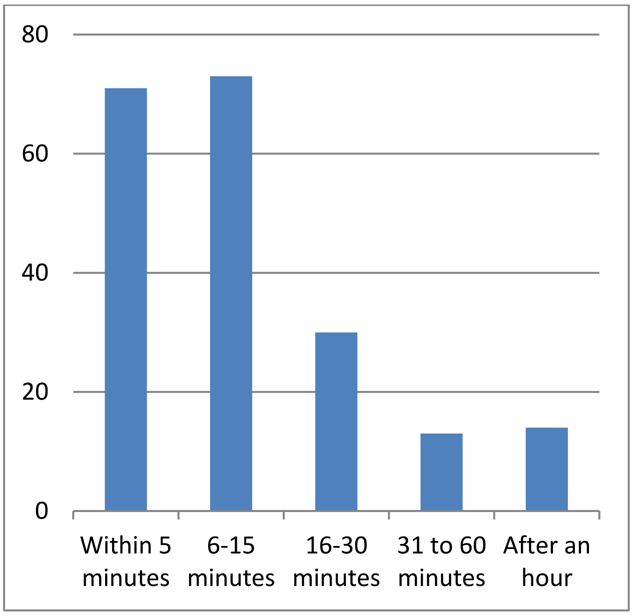

How soon after waking do you usually smoke now? (Qu. B14)

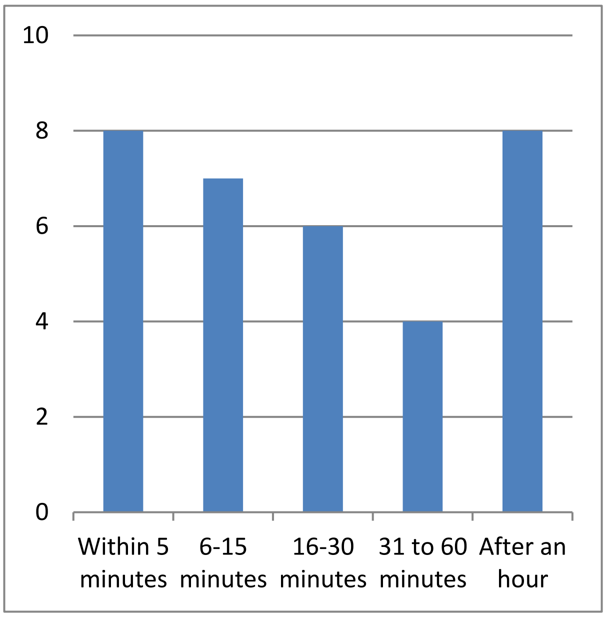

How did you stop smoking? (Qu. B19)

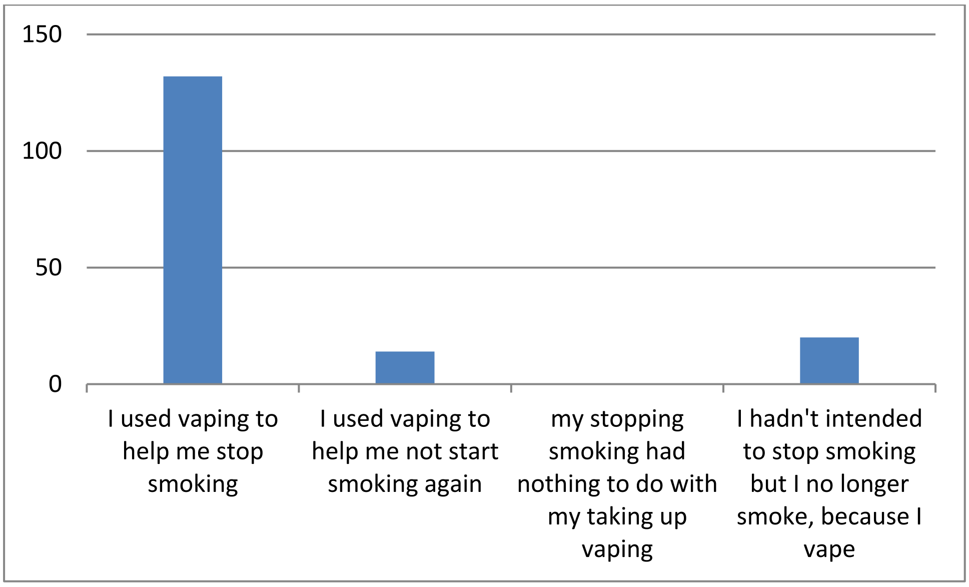

International Journal of
Environmental Research and Public Health
MDPI

C Please tell us a little more about yourself
1. Gender (Male, Female)
2. Age (Under 20, 20-25, 26-30, 31-35, 36-40, 41-45, 46-50, 51-55, 56-60, 61-65, 66-70, over 70)
3. Ethnicity (Māori, Pacific, NZ European, Asian, other)
4. Which region of New Zealand do you live in?

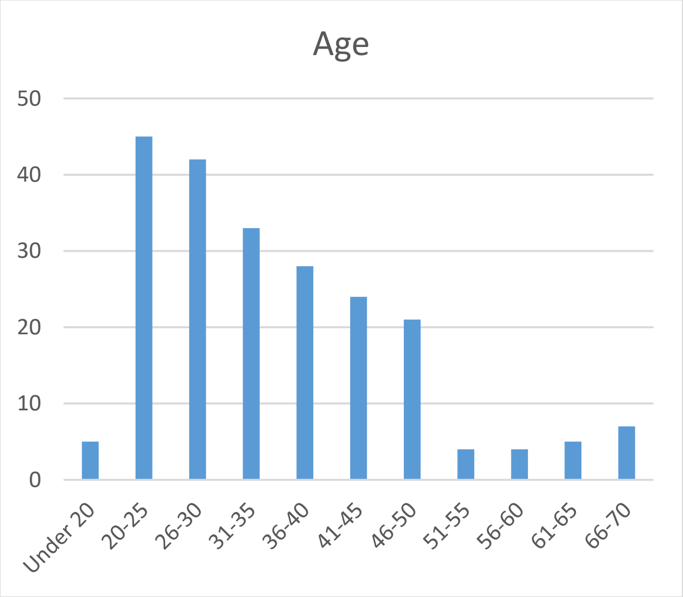

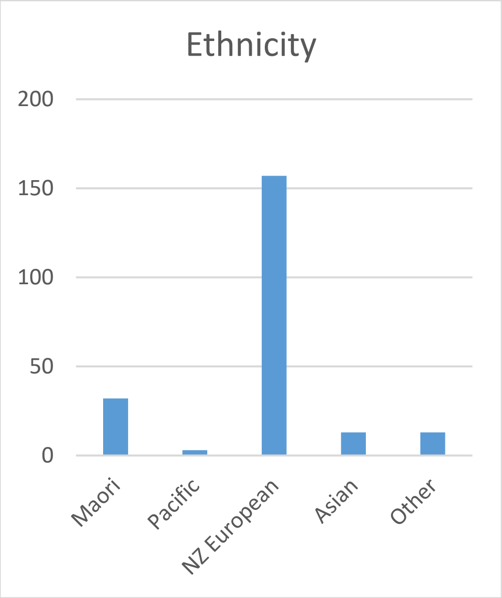

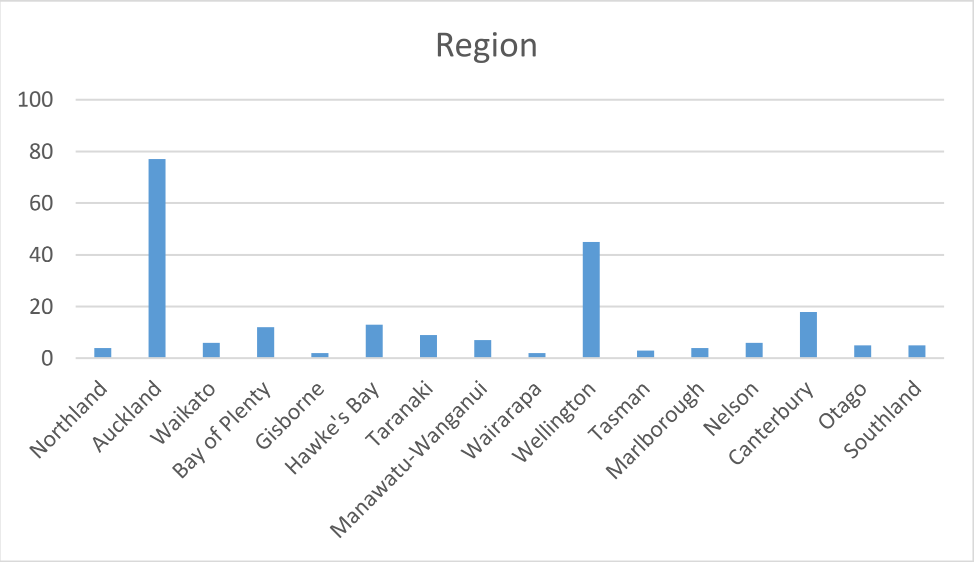

Supplement: Supplementary file 1 [file ijerph-15-00222-s001.zip › ijerph 254738-Supplementary Materials- File 1.pdf]
