# Supplementary figures and images for: An Online Survey of New Zealand Vapers
Source: Int J Environ Res Public Health. 2018 Jan 29;15(2):222. doi: 10.3390/ijerph15020222 (PMC5858291; doi:10.3390/ijerph15020222)

# Vaping Starts: N=218

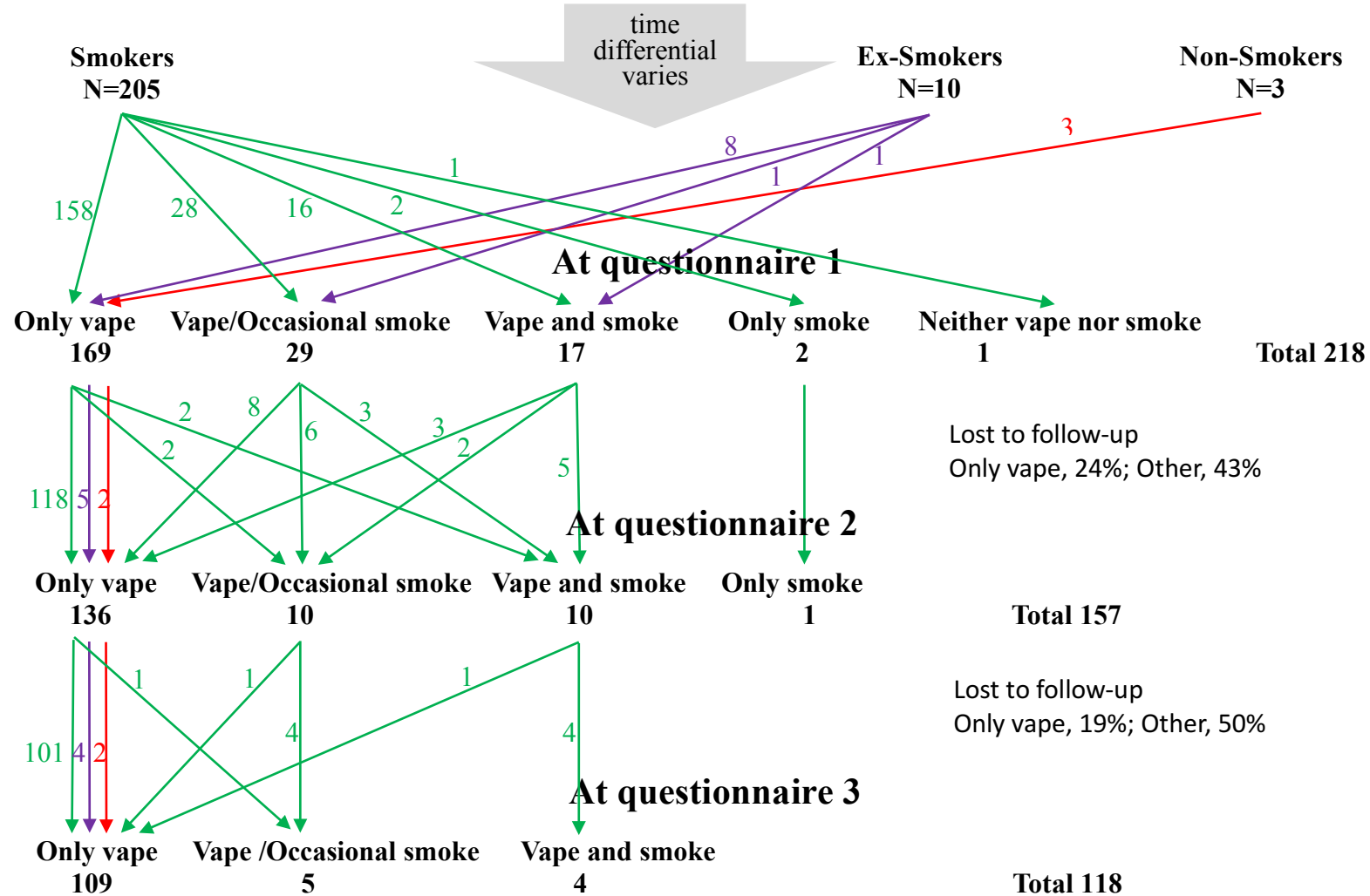

Supplement: Supplementary file 1 [file ijerph-15-00222-s001.zip › ijerph 254738 Supplementary Materials- File 3.pdf]
